# H19 Promotes Osteoblastic Transition by Acting as ceRNA of miR-140-5p in Vascular Smooth Muscle Cells

**DOI:** 10.3389/fcell.2022.774363

**Published:** 2022-02-07

**Authors:** Feng Xu, Jia-Yu Zhong, Bei Guo, Xiao Lin, Feng Wu, Fu-Xing-Zi Li, Su-Kang Shan, Ming-Hui Zheng, Yi Wang, Qiu-Shuang Xu, Li-Min Lei, Chang-Ming Tan, Xiao-Bo Liao, Ling-Qing Yuan

**Affiliations:** ^1^ National Clinical Research Center for Metabolic Diseases, Hunan Provincial Key Laboratory for Metabolic Bone Diseases, and Department of Metabolism and Endocrinology, The Second Xiangya Hospital of Central South University, Changsha, China; ^2^ Department of Nuclear Medicine, Xiangya Hospital of Central South University, Changsha, China; ^3^ Department of Radiology, The Second Xiangya Hospital of Central South University, Changsha, China; ^4^ Department of Pathology, The Second Xiangya Hospital of Central South University, Changsha, China; ^5^ Department of Cardiothoracic Surgery, The Second Xiangya Hospital of Central South University, Changsha, China

**Keywords:** H19, miR-140-5p, Satb2, smooth muscle cell differentiation, arterial calcification

## Abstract

Arterial medial calcification is a common disease in patients with type 2 diabetes, end-stage renal disease and hypertension, resulting in high incidence and mortality of cardiovascular event. H19 has been demonstrated to be involved in cardiovascular diseases like aortic valve diseases. However, role of H19 in arterial medial calcification remains largely unknown. We identified that H19 was upregulated in *ß*-glycerophosphate (*β*-GP) induced vascular smooth muscle cells (VSMCs), a cellular calcification model *in vitro*. Overexpression of H19 potentiated while knockdown of H19 inhibited osteogenic differentiation of VSMCs, as demonstrated by changes of osteogenic genes Runx2 and ALP as well as ALP activity. Notably, H19 interacted with miR-140-5p directly, as demonstrated by luciferase report system and RIP analysis. Mechanistically, miR-140-5p attenuated osteoblastic differentiation of VSMCs by targeting Satb2 and overexpression of miR-140-5p blocked H19 induced elevation of Satb2 as well as the promotion of osteoblastic differentiation of VSMCs. Interestingly, over-expression of Satb2 induced phosphorylation of ERK1/2 and p38MAPK. In conclusion, H19 promotes VSMC calcification by acting as competing endogenous RNA of miR-140-5p and at least partially by activating Satb2-induced ERK1/2 and p38MAPK signaling.

## Introduction

Arterial medial calcification (AMC) is a common cardiovascular disease, especially in patients with type 2 diabetes mellitus (T2DM), end-stage renal disease (ESRD), obesity-related disease state and ageing (Sarnak et al., 2019; Bourron et al., 2020). Nowadays, AMC has growing incidence because of the elevated aging population worldwide (McClelland et al., 2006). The epidemiologic data have demonstrated that the occurrence of AMC is an important risk factor for incidence and mortality of cardiovascular event (Budoff et al., 2019). In the past decades, AMC used to be thought as a negative and degenerative process resulting from deposition of hydroxyapatite in the arterial walls because of aging. However, with the advent of recent advance, AMC has now been illustrated to be a biological regulated active process that mainly involve the osteogenic transition of vascular smooth cell phenotype, resembling the process of osteoblast differentiation of pre-osteoblast cells (Xu et al., 2020). Notably, there exists significant ectopic expression of osteogenic phenotype genes such as alkaline phosphatase (ALP), runt related transcription factor 2 (Runx2), osteocalcin (OC), and bone morphogenetic protein 2 (BMP2) in calcified arterial walls (Rashdan et al., 2020; Xu et al., 2020). However, the underlying mechanism of AMC remains largely unknown and treatment strategies are limited.

H19, a well-known imprinted gene derived long non-coding RNA (lncRNA), was initially observed in liver and embryonic tissues as a key regulator of growth and development (Zhou et al., 2019). However, with the incoming brand-new various evidence, H19 has now been demonstrated to be widely involved in various physiological and pathological processes, such as stem cell development (Zhou et al., 2019), skeletal muscle differentiation and regeneration (Dey et al., 2014), cholestatic liver fibrosis (Song et al., 2017), aging and cancer progression (Lee et al., 2015). Recent advances have shown that increased circulating H19 level is closely related to higher risk of coronary artery diseases (Zhang et al., 2017; Bitarafan et al., 2019). A more recent study shown that H19 was increased in calcific aortic valve disease and promoted mineralization of valve interstitial cells (Hadji et al., 2016). However, the role of H19 in AMC is still largely unknown.

MicroRNAs (miRNAs) are a class of small non-coding RNAs (20–22 nucleotides) and has been demonstrated to affect gene expression by binding to 3′ untranslated region (3’ UTR) of target genes. A growing body of evidence have also demonstrated that lncRNAs may exert their biological functions by acting as competitive endogenous RNA (ceRNA) to sponge specific miRNAs (Thomson and Dinger, 2016). For example, H19 antagonized the expression of miRNA let-7 to increase muscle differentiation (Kallen et al., 2013). Similar effects were reported in miR-106a (Imig et al., 2015) and miR-194-5p (Li et al., 2018), et al. Intriguingly, miR-675, embedded in H19 exon 1, is the production of H19 transcript and has been demonstrated in mediating functions of H19 in placental growth, BMSCs differentiation and bladder cancer proliferation (Liu et al., 2016a).

Satb2, short for special AT-rich sequence-binding protein 2, is a novel identified DNA binding transcription factor that plays an important role in tissue development, such as bone regeneration, and cancer progression (Dobreva et al., 2006; Ni et al., 2021). Indeed, some studies have demonstrated that Satb2 shares similar pathway with Runx2 and works together to induce bone formation (Dobreva et al., 2006; Hu et al., 2015). More recently, Hao et al. found that down-regulation of satb2 decreased VSMC calcification, suggesting satb2 might be pro-calcification factor in VSMC calcification (Hao et al., 2016).

In the present study, we characterize the role H19 in AMC. The results showed that H19 was elevated in calcified vascular smooth muscle cells (VSMCs). Knockdown of H19 increased while overexpression of H19 decreased the osteogenic differentiation of VSMCs. Mechanistically, H19 increased the expression of Satb2 through sponging miR-140-5p. These data suggest that H19 promotes VSMC calcification by acting as competing endogenous RNA of miR-140-5p to increase the expression of Satb2.

## Materials and Methods

### Reagents

The fetal bovine serum (FBS), DMEM and penicillin/streptomycin mixture were purchased from Gibco-BRL Co., Ltd (Grand Island, NY, United States). Antibodies for Satb2 (sc-81376, 1:1,000), phospho-p38 MAPK (sc-166182, 1:1,000), p38 MAPK (sc-81621, 1:1,000), phospho-ERK1/2 (sc-81492, 1:1,000), and ERK1/2 (sc-135900, 1:1,000) were purchased from Santa Cruz Biotechnology Inc (Waltham, MA, United States). Runx2 (ab23981, 1:1,000) and ALP (ab229126, 1:1,000) antibodies were purchased from Abcam (Cambridge, United Kingdom). Anti-alpha smooth muscle Actin (α-SMA) antibody (bs-10196R, 1:1,000) was bought from Beijing Biosynthesis Biotechnology CO., LTD. (Beijing, China). Horseradish peroxidase (HRP)-conjugated Goat Anti-Mouse IgG, HRP-conjugated Goat Anti-Rabbit IgG as well as anti-GAPDH monoclonal antibody were purchased from Proteintech (Rosemont, United States). Satb2 specific siRNA oligonucleotides and the control siRNA oligonucleotides were purchased from Shanghai GenePharma Co., Ltd (Shanghai, China). MiR-140-5p mimics, miR-140-5p inhibitor, the control mimics, the control inhibitor, H19 specific smart silence and the control smart silence were purchased from Ribobio (Guangzhou, China). H19-expressing lentivirus were constructed based on Ubi-MCS-SV40-EGFP system (sequence based on NR_002196, gene ID: 283120) by Shanghai Genechem Co., Ltd (Shanghai, China). Satb2-expressing lentivirus (sequence based on NM_001172509) were bought from Shanghai Genechem Co., Ltd (Shanghai, China). The ALP assay kits were purchased from Nanjing Jiancheng Bioengineering Institute (Nanjing, China). Alizarin Red S and *ß*-glycerophosphate (β-GP) were purchased from Sigma Chemical Co., Ltd (St.Louis, MO, United States).

### Cell Culture

Human VSMCs were acquired and identified by a previously established method (Xu et al., 2019). Human VSMCs from excess donor normal arterial tissues after kidney transplantations were grown, which was approved by the Ethics Committee of the Second Xiangya Hospital of Central South University, China. Briefly, the tissue was fragmented (1,2 mm^3^), the aortas were minced and digested in digestion solution (0.125 mg/ml elastase, 0.25 mg/ml soybean trypsin inhibitor, 10 mg/ml collagenase I, 2.0 mg/ml crystallized bovine albumin, and 15 mM HEPES) at 37°C for 45 min. The cellular digests were filtered through a sterile 100 mM nylon mesh, centrifuged at 1,000 rpm for 10 min, and then washed twice in DMEM supplemented with 10% FBS and 1% penicillin/streptomycin before cultured in the same medium. All cells were incubated at 37°C with a humidified atmosphere containing 5% CO2. Cells passaged between 3 and 6 times were chosen for the experiments. VSMCs were demonstrated by typical peak-valley phenotype and expression of SMC marker *a*-SMA. Free of mycoplasma infection in VSMCs was confirmed by mycoplasma testing kit (Shanghai Seo Biotechnology Co., Ltd). Medium containing 10 mM *ß*-GP was used to induce osteoblastic differentiation of VSMCs as reported previously (Xu et al., 2019).

### Quantitative RT-PCR

Cells were incubated with TRIzol reagent (Invitrogen, Carlsbad, CA, United States) and total RNA was extracted as guided by the manufacturer’s instructions (Wu et al., 2020). For detection of mRNA and LncRNA expression, cDNA was synthesized with the guidance of the PrimeScript RT reagent Kit (Takara, Japan) and then Real-time PCR detection was performed by using the TB Green™ Premix Ex Taq™ II kit (Takara, Japan) with GAPDH as normalized control. For miRNA detection, miRNA was re-transcribed and analysed by All-in-One™ miRNA quantitative RT-PCR Detection System (GeneCopoeia, Rockville, United States) with guidance by the manufacturer’s protocol and using U6 as the normalized control. H19 primers (forward: 5- GCG​TCC​GGC​CTT​CCT​GAA​C-3, reverse: 5-GAG​CTG​GGT​AGC​ACC​ATT​TCT​T-3) were as designed and synthesized by Shanghai Genechem Co., Ltd (Shanghai, China). U6 (HmiRQP9001), miR-140-5p (HmiRQP0181), miR-675-5p (HmiRQP0782), miR-22-3p (HmiRQP0332), miR-17-3p (HmiRQP0229), miR-29b-3p (HmiRQP0373), miR-106b-5p (HmiRQP0029), miR-214-5p (HmiRQP0321), GAPDH (HQP006940), Satb2 (HQP096946), ALP (HQP006440), and Runx2 (HQP016478) primers were purchased from GeneCopoeia.

### Western Blot

Proteins analysis by western blot was performed in the same way as described previously (Liang et al., 2012; Peng et al., 2017; Lin et al., 2020). Briefly, total protein were lysed by using of RIPA lysate (Beyotime Biotechnology, Shanghai, China) supplemented with cocktail protease inhibitor (Sellect, Shanghai, China) and the extraction was measured by BCA Protein Assay kit (Beyotime Biotechnology, Shanghai, China). Total protein were then separated by 12% SDS-PAGE and transferred to 0.2 µm Polyvinylidene fluoride Membranes (PVDF, Millipore, Billerica, MA). The membranes were blocked by 5% milk for 1 h at room temperature, followed by incubation with specific primary antibody overnight. And then the blots were rewarmed to room temperature for 30 min, washed by phosphate-buffered saline (PBS) for 3 times with 10 min interval and followed by incubation with HRP-labeled secondary antibody for 1 h at room temperature. Finally, the reaction was detected by chemiluminescence assay with Luminata™ Crescendo Western HRP Substrate (Millipore, Billerica, MA) and was analyzed by ABI system.

### Analysis of the ALP Activity

The cell layers were subjected to a lysis solution containing 20 mM Tris–HCl, pH 8.0, and 150 mM NaCl, 1% Triton X-100, 0.02% NaN_3_ and 1 mM PMSF. And then the lysates were sonicated for 4 times with 5 s interval. The ALP activity was measured by detecting *p*-nitrophenol release using the ALP kit (Liu et al., 2016b). The ALP activity was normalized by the total protein level of the cell lysate.

### Detection of Alizarin Red S. Staining Level

Alizarin Red S. staining was conducted in a similar way as described previously (Liao et al., 2013). Briefly, VSMCs were cultured in 24-well plates for indicated time. Then, the content of mineralized matrix was examined by Alizarin Red S staining. Briefly, cells were fixed in 70% ethanol for 1 h at room temperature and then stained with 40 mM Alizarin Red S for 10 min. Next, cells were washed with PBS for 3 times to reduce nonspecific staining. The stained matrix was digitally photographed.

### Luciferase Reporter Gene Assay System

The fragment of the wild type or mutant Satb2 3′ UTR was ligated into a pmirGLO -dual-luciferase miRNA target expression vector (Promega, United States). The constructed vectors were then identified by sequencing, cloned, and purified. VSMCs were treated with co-transfection of the luciferase reporter system that carried wild-type Satb2 3′ UTR sequence, mutant Satb2 3′ UTR sequence, and miR-140-5p mimics or scramble oligonucleotides. For detection of interaction between H19 and miR-140-5p, plasmid with wild-type H19 (with binding site to miR-140-5p) or mutant H19 sequence (with mutant binding site to miR-140-5p) were co-transfected with miR-140-5p mimics or scramble control. Subsequently, luciferase activities were determined by the luciferase assay system (Promega) at 48 h after transfection in accordance with the manufacturer’s protocol. The ratio of firefly to Renilla was used to normalize the firefly luciferase values. The specific primers for synthesis of wild type or mutant 3′UTR Satb2 mRNA, or wild type or mutant H19 were purchased from Ribobio.

### RNA-Binding Protein Immunoprecipitation (RIP) Assay

RIP experiments were performed using the Magna RIP™ Kit (Millipore, Billerica, MA). Briefly, VSMCs were treated with magnetic beads that were conjugated with anti-Ago2 antibody (Cambridge, United Kingdom) or negative control IgG (Cambridge, United Kingdom). Thereafter, the beads with conjugated molecules were treated by protein A/G beads and H19 and miR-140-5p levels were determined by qRT-PCR analysis.

### Statistical Analysis

SPSS 23.0 (SPSS, Chicago, IL, United States) was used for all statistical comparisons. Data are presented as means ± standard deviation (SD) unless specially stated. Student’s *t* test was performed when comparing two groups. One-way or two-way ANOVA followed by the Tukey’s HSD post hoc analysis was performed for multiple comparisons. Differences at *p* value under 0.05 were considered statistically significant.

## Results

### H19 Promotes Osteoblastic Differentiation and Mineralization of VSMCs

**FIGURE 1:**
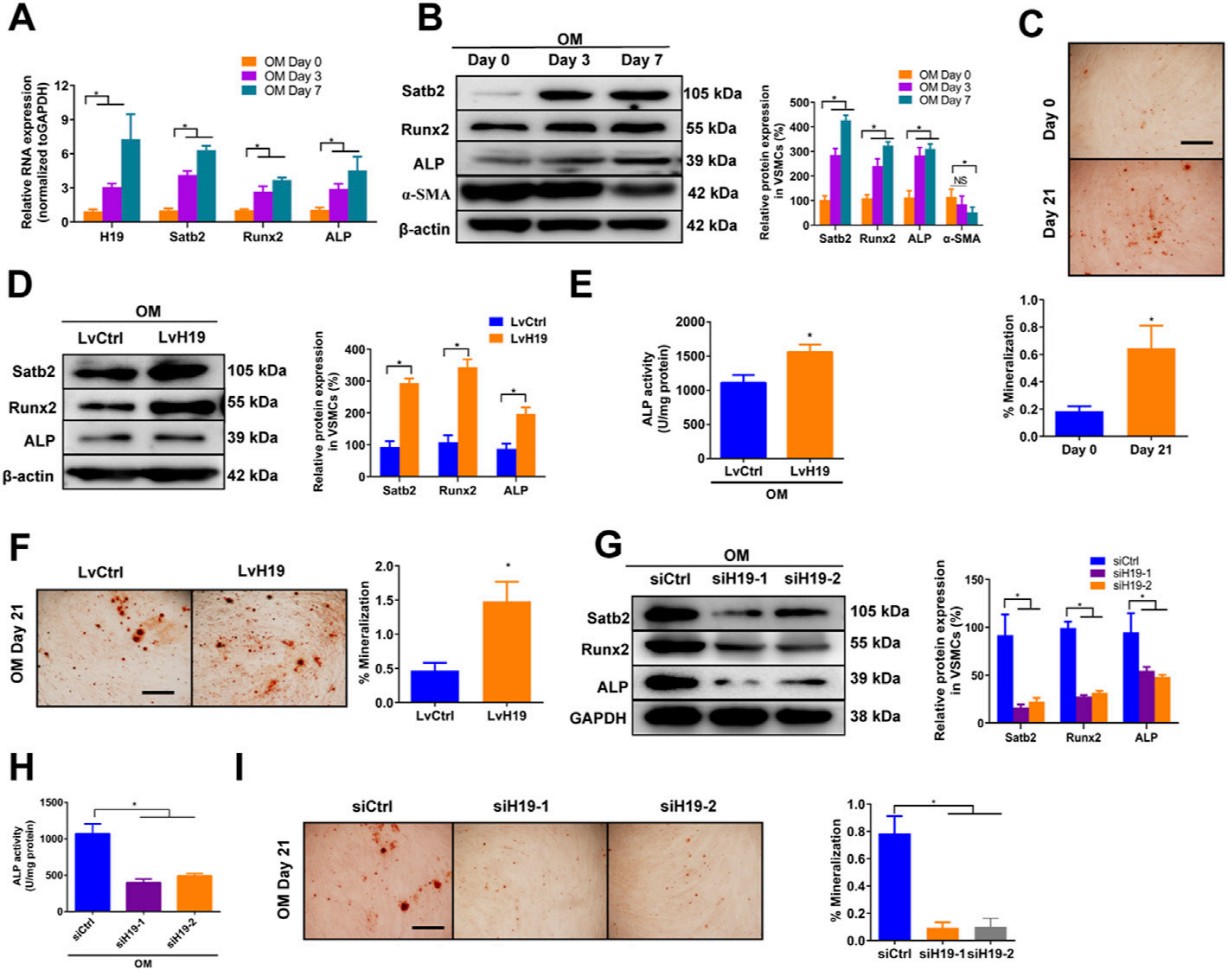
| H19 promotes osteogenic differentiation and mineralization of VSMCs treated with osteogenic medium. **(A,B)** Expression of H19, calcification marker genes Runx2 and ALP, and Satb2 during the osteogenic differentiation of VSMCs. VSMCs were treated with osteogenic medium (OM) that contains 10 mM *ß*-glycerophosphate (*β*-GP) for 7 days to induce osteogenic differentiation. **(A)** Expression of H19, Runx2, ALP, and Satb2 were determined by qRT-PCR. **(B)** Expression of Runx2, ALP, *a*-SMA and Satb2 were determined by Western blotting. The data were presented as densitometric ratios normalized to *ß*-actin [**(B)**, right panel]. **(C)** Alizarin Red S staining were measured in VSMCs incubated with *ß*-GP for 21 days. Representative microscopic views are shown. Data were presented as ratio of positive staining area (lower panel). **(D–F)** VSMCs were treated with OM along with either lentivirus containing full length of H19 cDNA (LvH19) or control cDNA (LvCtrl). The protein expression of Runx2, ALP, and Satb2 were determined by Western blotting in VSMCs after treatment for 2 days **(D)**. Data were presented as densitometric ratios normalized to GAPDH [**(D)**, right panel]. The ALP activity was determined by an Elisa kit in VSMCs after treatment 2 days **(E)**. Alizarin Red S staining were measured in VSMCs after treatment for 21 days. Representative microscopic views are shown **(F)**. Scale bar represents 200 µm. Data were presented as ratio of positive staining area [**(F)**, right panel]. **(G–I)** VSMCs were incubated with OM medium along with either H19 siRNA (siH19) or control siRNA (siCtrl). The protein expression of Runx2, ALP, and Satb2 were determined by Western blotting after treatment for 2 days **(G)**. Data were presented as densitometric ratios normalized to GAPDH [**(G)**, right panel]. The ALP activity was determined by an Elisa kit in VSMCs after treatment for 2 days **(H)**. Alizarin Red S staining were measured in after treatment for 21 days. Representative microscopic views are shown **(I)**. Scale bar represents 200 µm. Data were presented as ratio of positive staining area [**(I)**, right panel]. Each experiment was repeated for three times. The data represent the mean ± SD of triplicates. *p < 0.05.

To assess the expression of H19 during the osteoblastic differentiation of VSMCs, we firstly used osteogenic medium (OM) -induced VSMCs that contained 10 mM *ß*-GP as *in vitro* calcification model and then observed the change of H19 and calcification markers such as Runx2 and ALP as well as Satb2. In the control medium (CM) treated VSMCs, no obvious change of H19 expression in VSMCs among Day 0, 3 and 7 (Supplementary Figure S1A). Similar results were found in both mRNA and proteins level of osteogenic marker such as Runx2 and ALP as well as Satb2 (Supplementary Figures S1A,B). In addition, contractile marker *a*-SMA did not change over time (Supplementary Figure S1B). Moreover, no difference in Alizarin Red S staining level in CM-treated VSMCs for Day 0 and Day 21 (Supplementary Figure S1C). However, Runx2 and ALP as well as Satb2 were increased but *a*-SMA was reduced in VSMCs after the treatment of *ß*-GP (Figures 1A,B), accompanied with increased Alizarin Red S Staining level (Figure 1C). These data suggest the successful establishment of *in vitro* calcification model. Moreover, the expression of H19 were increased significantly in OM-induced VSMCs in a time-dependent manner (Figure 1A), suggesting that H19 may be involved in osteoblastic differentiation of VSMCs.

Next, we determined whether H19 regulated VSMCs calcification by using loss- and gain-function of H19 (Supplementary Figures S2A,B). Overexpression of H19 by lentivirus containing H19 sequence (LvH19) resulted in increased protein expression of Runx2, ALP and Satb2, and ALP activity as well as Alizarin Red S staining level in both CM-treated VSMCs and OM-treated VSMCs when compared with control group (Figures 1D–F and Supplementary Figures S3A–C). In contrast, siRNA-mediated knockdown of H19 (siH19) decreased the expression of Runx2, ALP and Satb2 as well as ALP activity in OM-treated VSMCs when compared with control group (Figures 1G,H). Moreover, Alizarin Red S staining level of VSMCs were also decreased after knockdown of H19 (Figure 1I). In CM-treated VSMCs, reduced expression of Satb2 and Runx2 were also observed but no difference was found in ALP expression as well as ALP activity (Supplementary Figures S3D,E). Notably, due to the low base line lever, reduced Alizarin red S staining was observed but with no significance after down-regulation of H19 (Supplementary Figure S3F). Taken together, these data indicate that H19 promotes osteoblastic differentiation and mineralization of VSMCs.

### H19 Interacts With miR-140-5p

**FIGURE 2:**
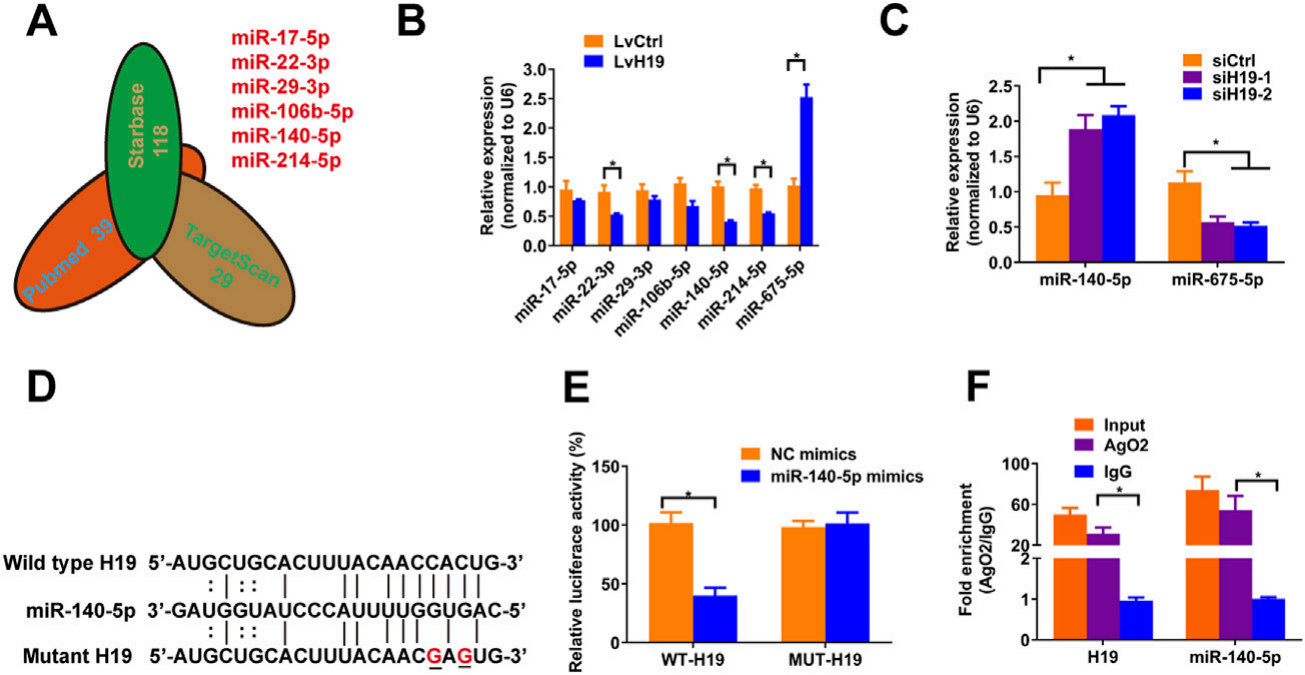
H19 interacts with miR-140-5p. **(A)** Predication of potential targets of H19 by using of bioinformatic analysis methods starbase and Targetscans in combination with Pubmed database. The number indicated potential miRNAs that may inhibit osteogenic differentiation of VSMC or interact with H19. **(B)** VSMCs were treated with lentivirus containing full length of H19 cDNA (LvH19) or control cDNA (LvCtrl). qRT-PCR was performed to evaluate expression of miR-17-5p, miR-22-3p, miR-29-3p, miR-106b-5p, miR-140-5p, miR-214-5p, and miR-675-5p in VSMCs transfected with LvH19 or LvCtrl. **(C)** The expression of H19 in VSMCs after infected with H19 siRNA (siH19) or control siRNA (siCtrl). Expression of miR-140-5p and miR-675-5p were evaluated by qRT-PCR in VSMCs infected with siH19 or siCtrl. **(D)** Schematic view of miR-140-5p putative target sites in WT and MUT H19 3′-UTR. **(E)** VSMCs were transfected with luciferase report vector containing WT or MUT H19 3′-UTR following by transfection of miR-140-5p mimics or control mimics. Luciferase activity was determined 48 h post transfection and luciferase activity was normalised to Renilla luciferase activity. **(F)** RIP analysis was performed to evaluate enrichment of H19 and miR-140-5p in Ago2 immunoprecipitates. Each experiment was repeated for three times. The data represent the mean ± SD of triplicates. *p < 0.05.

To verify the mechanism by which H19 regulates VSMCs calcification, we screened Pubmed databases for 39 miRNAs that were reported to be associated with osteoblastic differentiation of BMSCs and VSMCs. Then we assessed the potential interaction between these miRNAs and H19 by using starbase databases. Thereafter, we used TargetScan (Version 7.2) to confirm miRNAs that have conserved binding sequence with Runx2, BMP2 and Satb2 (Figure 2A). We selected miR-17a-5p, miR-22-3p, miR-29-3p, miR-106b-5p, miR-140-5p, and miR-214-5p for further analysis. Then, we assessed the expression of these miRNAs after overexpression of H19. The results shown that overexpression of H19 increased the expression of miR-675-5p, which has been demonstrated to be H19-derived miRNAs, and decreased the expression of miR-22-3p, miR-140-5p and miR-214-5p in VSMCs, with the most significant reduction in miR-140-5p (Figure 2B). In contrast, knockdown of H19 increased the expression of miR-140-5p in VSMCs (Figure 2C). Importantly, luciferase activity reporter assay system revealed that transfection of miR-140-5p mimics significantly decreased luciferase activity in wild type H19 but not mutant H19 that was devoid of conserved miR-140-5p binding site (Figures 2D,E). Interestingly, RIP analysis also shown enrichment of H19 and miR-140-5p in Ago2 pull-down precipitate (Figure 2F), suggesting miR-140-5p might mediate degradation of H19. These results suggest that H19 functions as a “sponge” of miR-140-5p in VSMCs.

### MiR-140-5p Restrains VSMCs Calcification by Targeting Satb2

**FIGURE 3:**
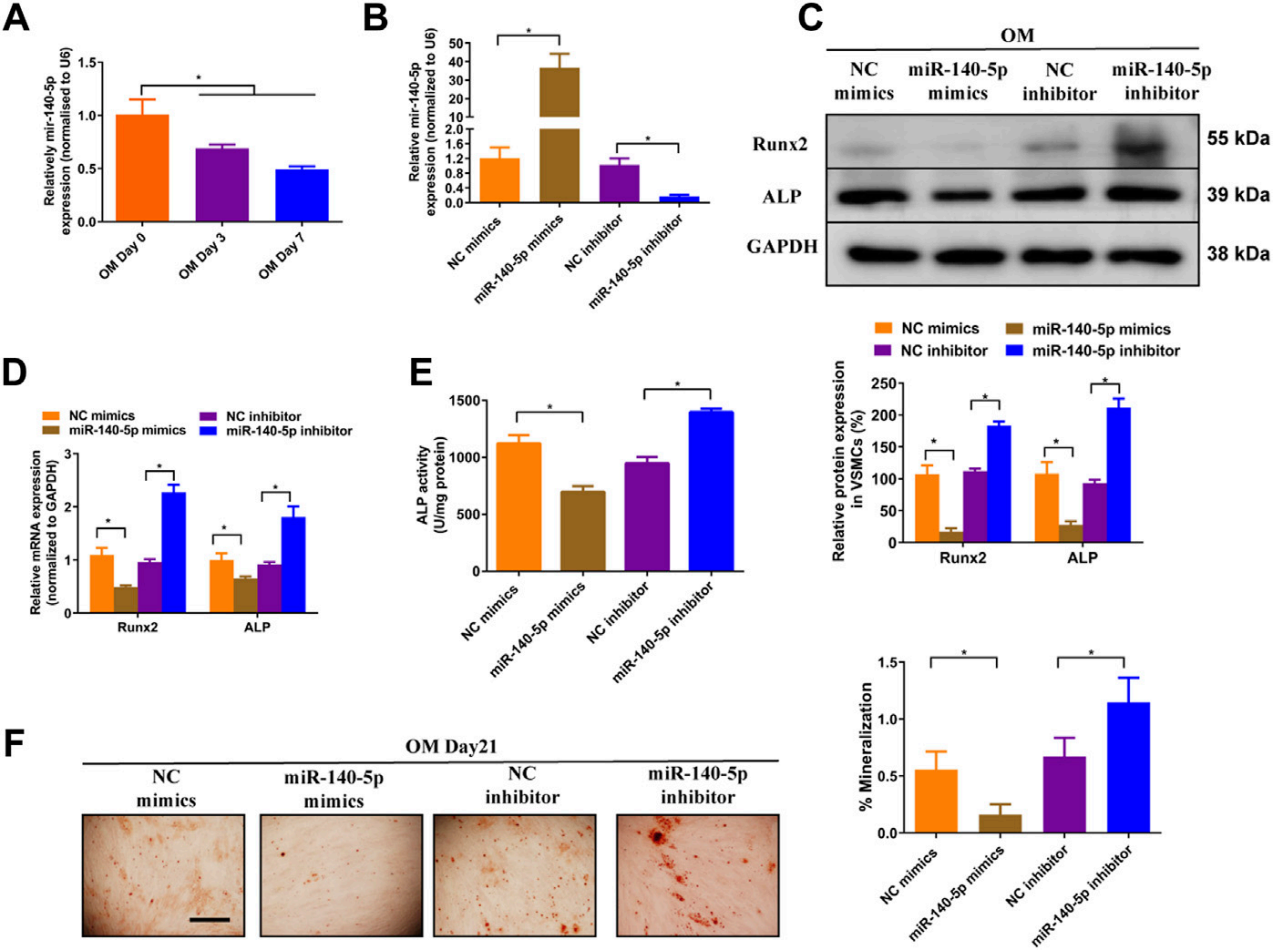
miR-140-5p antagonises osteogenic differentiation of VSMCs. **(A)** qRT-PCR was performed to evaluate the expression of miR-140-5p in VSMCs cultured in OM medium. **(B)** qRT-PCR was performed to evaluate the expression of miR-140-5p in VSMCs transfected with specific miR-140-5p mimics or inhibitor. **(C)** Western blotting was performed to determine the protein expression of Runx2 and ALP in VSMCs transfected with specific miR-140-5p mimics or inhibitor. The data were presented as densitometric ratios normalized to GAPDH [**(C)**, lower panel]. **(D)** qRT-PCR was performed to determine the protein expression of Runx2 and ALP in VSMCs transfected with specific miR-140-5p mimics or inhibitor. **(E)** The ALP activity was evaluated by specific Elisa kits in VSMCs transfected with specific miR-140-5p mimics or inhibitor. **(F)** Alizarin Red S staining were measured in *ß*-GP-treated VSMCs transfected with specific miR-140-5p mimics or inhibitor for 21 days. Representative microscopic views are shown. Scale bar represents 200 µm. Data were presented as ratio of positive staining area [**(F)**, right panel]. Each experiment was repeated for three times. The data represent the mean ± SD of triplicates. *p < 0.05.

Previous studies have shown that miR-140-5p inhibited osteoblast differentiation of human BMSCs and bone development in zebrafish embryonic by targeting BMP2 (Hwang et al., 2014; Gan et al., 2016). However, role of miR-140-5p in VSMCs calcification is largely unknown. To assess the effects of miR-140-5p on osteoblastic differentiation of VSMCs, we firstly determined the expression of miR-140-5p in 10 mM *ß*-GP induced VSMCs. The results shown that miR-140-5p was decreased during the process of VSMCs calcification in a time-dependent manner (Figure 3A). Moreover, overexpression of miR-140-5p reduced the expression of Runx2 and ALP (Figures 3B–D) as well as ALP activity (Figure 3E). In contrast, down-regulation of miR-140-5p increased mRNA and protein expression of Runx2 and ALP (Figures 3B–D) as well as ALP activity (Figure 3E). Similar change was also found in VSMCs by using Alizarin Red S staining (Figure 3F). Taken together, these data validates the inhibition effects of miR-140-5p on VSMCs calcification.

**FIGURE 4:**
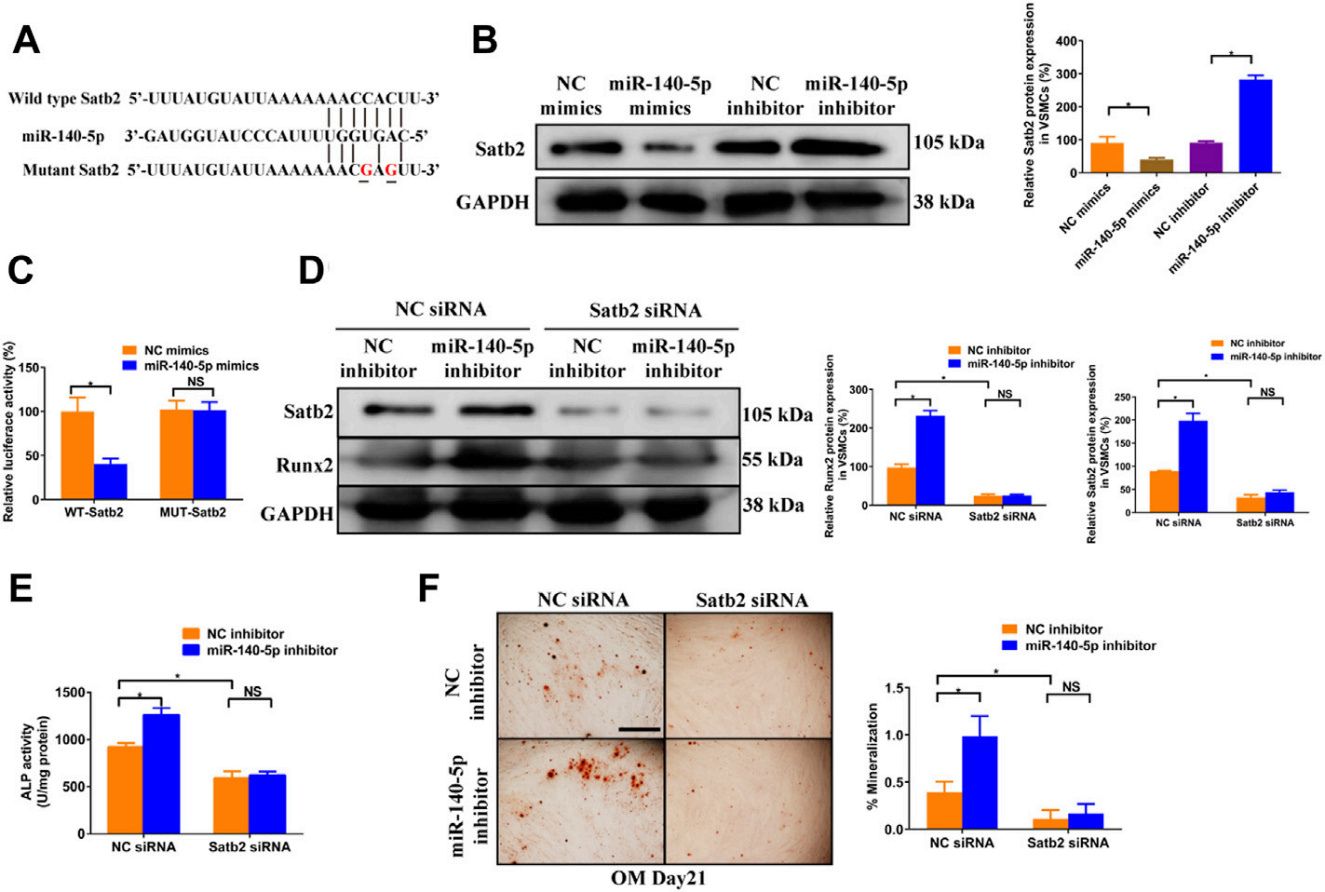
miR-140-5p antagonises osteogenic differentiation of VSMCs by targeting Satb2. **(A)** Schematic view of miR-140-5p putative binding sites in WT and MUT Satb2 3′-UTR. **(B)** Western blotting was performed to determine the protein expression of Satb2 in VSMCs transfected with specific miR-140-5p mimics or inhibitor. The data were presented as densitometric ratios normalized to GAPDH [**(B)**, lower panel]. **(C)** VSMCs were transfected with luciferase report vector containing WT or MUT Satb2 3′-UTR following by transfection of miR-140-5p mimics or control mimics. Luciferase activity was determined 48 h post transfection and luciferase activity was normalised to Renilla luciferase activity. **(D)** Western blotting was performed to determine the protein expression of Satb2 and Runx2 in VSMCs transfected with specific miR-140-5p inhibitor with or without Satb2 siRNA. The data were presented as densitometric ratios normalized to GAPDH [**(D)**, lower panel]. **(E)** The ALP activity was determined by an Elisa kit in VSMCs infected with inhibitor of miR-140-5p in the presence of Satb2 siRNA or not. **(F)** Alizarin Red S staining were measured in *ß*-GP-treated VSMCs infected with inhibitor of miR-140-5p in the presence of Satb2 siRNA or not for 21 days. Representative microscopic views are shown. Scale bar represents 200 µm. Data were presented as ratio of positive staining area [**(F)**, right panel]. Each experiment was repeated for three times. The data represent the mean ± SD of triplicates. *p < 0.05.

To gain insight into the mechanism by which miR-140-5p restrained VSMCs calcification, online bioinformatics tool TargetScan (version 7.2, http://www.targetscan.org/vert_72/) and miRDB (http://mirdb.org/mirdb/index.html) as well as miRmap (https://mirmap.ezlab.org/app/) were adopted to predict potential miR-140-5p targeting genes. Of these, Satb2 has been shown to possess properties as the key regulator for the process of bone information as well as VSMC calcification (Hao et al., 2016; Liu et al., 2020). Figure 4A illustrated the potential binding sites of miR-140-5p with Satb2, suggesting that Satb2 may be targeting gene of miR-140-5p. Furthermore, western blot showed that Satb2 protein level was down-regulated by miR-140-5p mimics and up-regulated by miR-140-5p inhibitor (Figure 4B). Luciferase report assay also demonstrated that overexpression of miR-140-5p reduced wild type Satb2 promotor activity but not that of mutant Satb2 (Figures 4A,C). These data imply that Satb2 may be the target of miR-140-5p in VSMCs. To make clear whether Satb2 mediates the inhibition effect of miR-140-5p on VSMCs calcification, we also used Satb2 specific siRNA to block its expression. Down-regulation of Satb2 reduced the expression of Runx2 and decreased ALP activity as well as Alizarin Red S staining (Figures 4D–F), suggesting that Satb2 plays a important role in VSMCs calcification. Of note, inhibition of miR-140-5p increased the expression of Runx2 and enhanced ALP activity as well as Alizarin Red S staining, but these effects were abolished by suppression of Satb2 (Figures 4D–F). Taken together, these results demonstrate that miR-140-5p inhibits VSMCs calcification by targeting Satb2.

**FIGURE 5:**
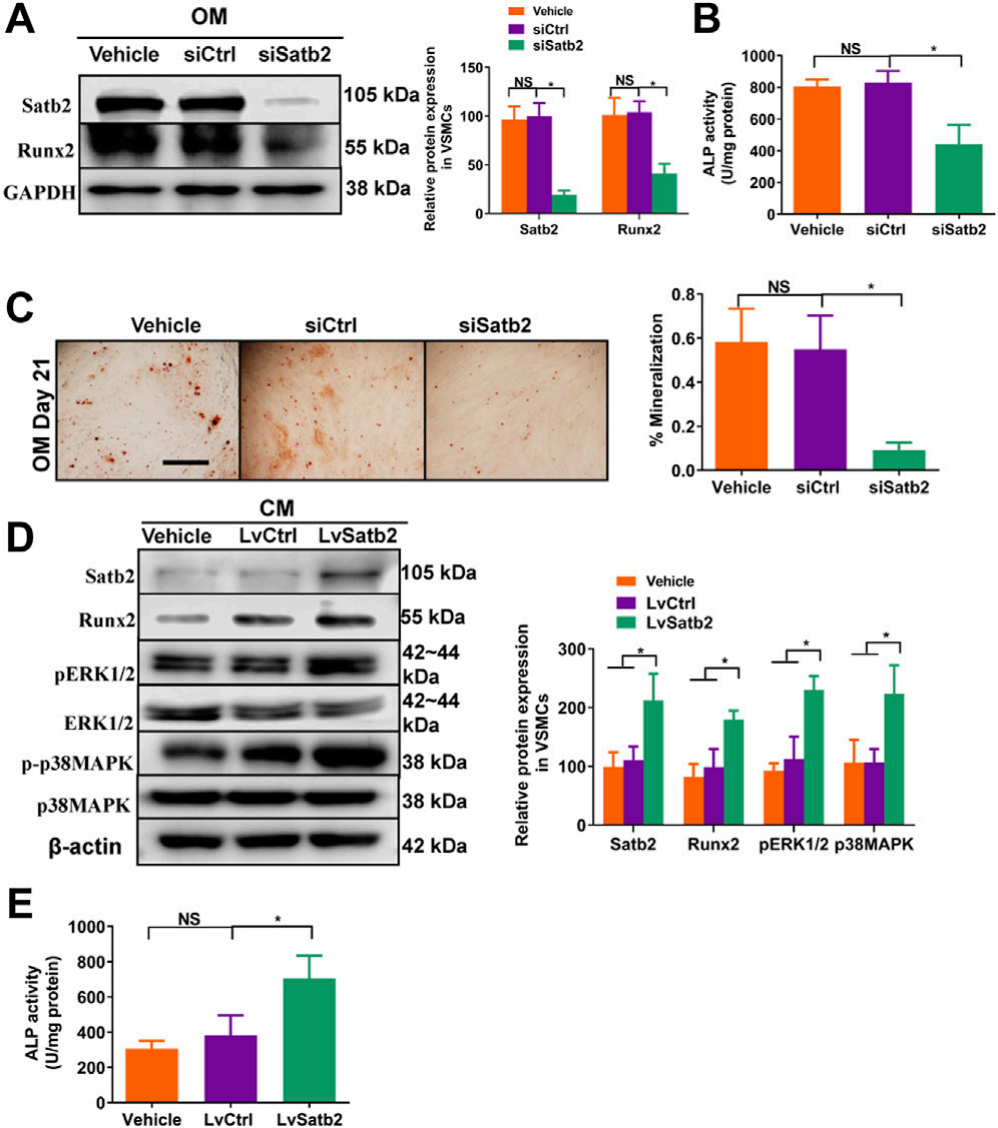
Knockdown of Satb2 inhibits osteogenic differentiation of VSMCs. OM-treated VSMCs were transfected with control siRNA (siCtrl), Satb2 siRNA (siSatb2), or vehicle control for 48 h. **(A)** Western blotting was performed to evaluate Satb2 and Runx2 expression. The data were presented as densitometric ratios normalized to GAPDH [**(A)**, right panel]. **(B)** The ALP activity was determined by an Elisa kit. **(C)** Alizarin Red S staining were measured in *ß*-GP-treated VSMCs infected with siCtrl or siSatb2 for 21 days. Representative microscopic views are shown. Scale bar represents 200 µm. Data were presented as ratio of positive staining area [**(C)**, right panel]. CM-treated VSMCs were infected with control lentivirus (LvCtrl), Satb2-expressing lentivirus (LvSatb2), or vehicle control for 48 h. **(D)** Western blotting was performed to evaluate Satb2, Runx2, pERK1/2, ERK1/2, p38MAPK, and p38MAPK expression. The data were presented as densitometric ratios normalized to *ß*-actin or phosphorylation/total protein [**(D)**, right panel]. **(E)** The ALP activity was determined by an Elisa kit. Each experiment was repeated for three times. The data represent the mean ± SD of triplicates. *p < 0.05.

To further validate the role of Satb2 on VSMC calcification, we determined the change of calcification markers after knockdown or over-expression of Satb2. Satb2 siRNA mediated down-regulation of Satb2 significantly reduced the expression of Runx2 and ALP activity as well as Alizarin Red S staining when compared with control group in OM-incubated VSMCs (Figures 5A–C). In addition, in CM-treated VSMCs, over-expression of Satb2 induced expression of Runx2 (Figure 5D) and increased ALP activity (Figure 5E). Interestingly, over-expression of Satb2 induced phosphorylation of ERK1/2 and p38MAPK (Figure 5D), two important kinases in cellular differentiation. Taken together, these data indicate that Satb2 is responsible for the process of VSMC calcification.

### H19 Enhances Osteoblastic Differentiation of VSMCs by Sponging miR-140-5p

**FIGURE 6:**
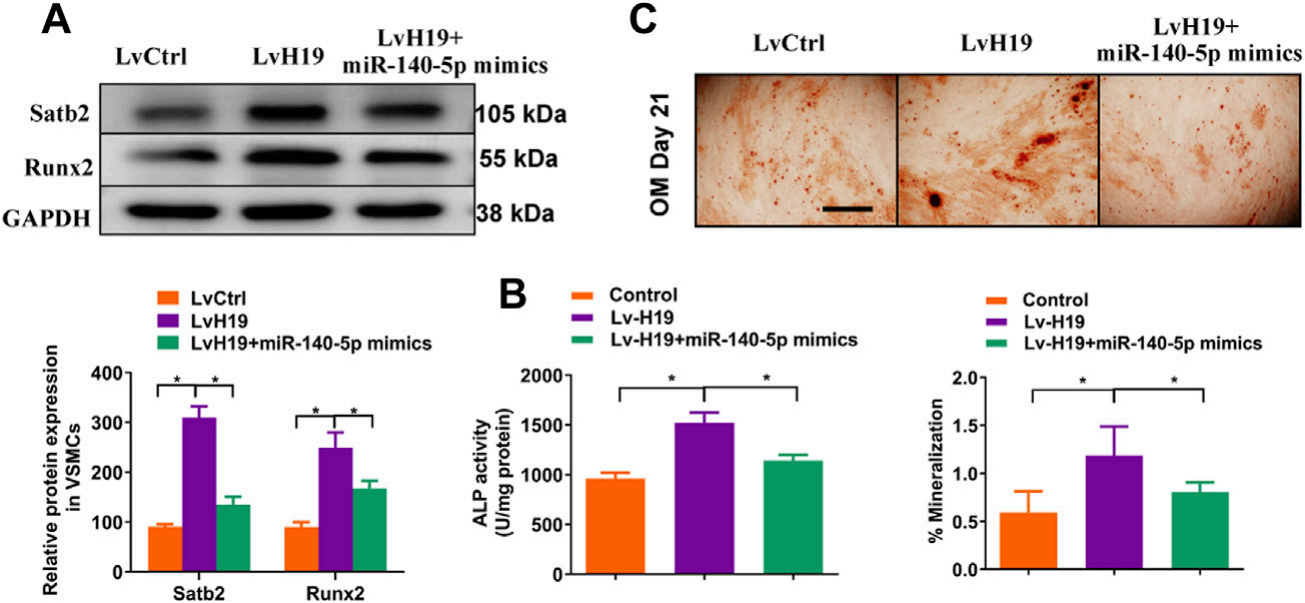
H19 promotes osteogenic differentiation of VSMCs by sponging miR-140-5p. VSMCs were transfected with LvH19, miR-140-5p, or corresponding negative control. **(A)** Western blotting was performed to evaluate Satb2 and Runx2 expression. The data were presented as densitometric ratios normalized to GAPDH [**(A)**, lower panel]. **(B)** The ALP activity was determined by an Elisa kit. **(C)** Alizarin Red S staining were measured in *ß*-GP-treated VSMCs infected with LvCtrl or LvH19 in the presence of miR-140-5p mimics or not for 21 days. Representative microscopic views are shown. Scale bar represents 200 µm. Data were presented as ratio of positive staining area [**(C)**, lower panel]. Each experiment was repeated for three times. The data represent the mean ± SD of triplicates. *p < 0.05.

To further validate the role of miR-140-5p in H19-induced calcification of VSMC, we upregulated the expression of miR-140-5p in VSMCs in the presence of H19. The results showed that overexpression of miR-140-5p partially abolished H19 induced expression of Runx2 (Figure 6A) and ALP activity (Figure 6B) as well as Alizarin Red S staining (Figure 6C), suggesting miR-140-5p is involved in H19 induced VSMC calcification. Interestingly, H19-induced expression of Satb2 was also partially blocked by overexpression of miR-140-5p (Figures 6A–C). These data suggest that H19 induces VSMC calcification via miR-140-5p/Satb2 axis.

## Discussion

**FIGURE 7:**
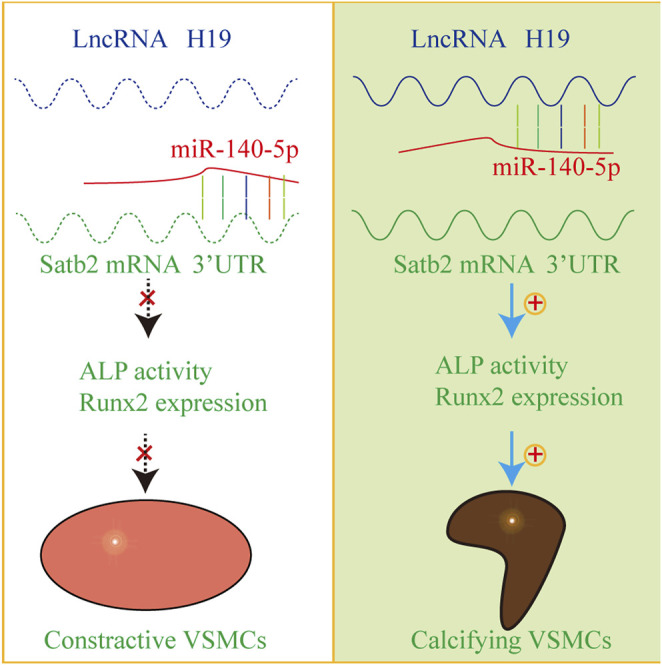
The mechanism diagram about H19/miR-140-5p/Satb2 axis in the regulation of osteogenic differentiation of VSMCs. Normally, miR-140-5p antagonizes the expression of Satb2 to inhibit osteogenic differentiation of VSMCs in absence of H19. However, overexpression of H19 can sponge miR-140-5p and release Satb2. As a result, VSMCs undergo phenotype transition into calcifying VSMCs and induce vascular calcification.

In the present study, we provide evidence that H19 plays an important role in the process of VSMC calcification. Over-expression of H19 increased while knockdown of H19 decreased the osteogenic phenotype transition of VSMC. Notably, H19 directly interacted with miR-140-5p and inhibition of miR-140-5p partially blocked H19-induced VSMC calcification. Taken together, these data show that H19 induces VSMC calcification by regulating miR-140-5p/Satb2 loop (Figure 7).

We firstly demonstrated that H19 induced osteogenic differentiation of human VSMCs. We reported that H19 was increased in calcifying VSMCs in a similar trend with calcification marker genes Runx2, which suggested that H19 may be associated with the process of VSMC calcification. Moreover, we found that over-expression of H19 promoted while downregulation of H19 alleviated VSMC calcification, suggesting that H19 play an essential role in VSMC calcification. Our results are in consistent with previous studies that knockdown of H19 reduced calcification of VSMCs (Liu et al., 2020). In their study, knockdown of H19 reduced phosphorylation of p38 and ERK1/2. However, how H19 regulates p38 MAPK and ERK is unclear. It seems that activation of p38 and ERK1/2 might be the common signaling for VSMC calcification but not specific for H19. Moreover, H19, a well-known long non-coding, acts mostly by long-term transcriptional regulation instead of rapid and transient signaling pathway. Mostly, activation of signaling pathway might by caused by classical ligand-receptor method. In our present work, however, we have demonstrated clear clues for H19-miR-140-5p-Satb2 loops in the regulation of VSMC calcification, which provides novel understanding that H19 regulate VSMC calcification by acting as the sponging RNA of miR-140-5p. Notably, we found that over-expression of Satb2 induced phosphorylation of p38 MAPK and ERK1/2, which suggest that Satb2 might be responsible for H19-induced activation of p38 MAPK and ERK1/2. However, it is unclear how Satb2 affects p38 MAPK and ERK signaling pathway.

By acting as a ceRNA to sponging miRNAs, H19 has been demonstrated to communicate with various miRNAs, including let-7, miR-17-5p, and miR-106a, to affect the expression of protein-coding mRNAs indirectly and thus regulate multiple physiological and pathological processes (Zhang et al., 2019). In consistent with these findings, we characterized that miR-140-5p interacted with H19 directly and inhibition of miR-140-5p abolished H19-induced VSMC calcification, as demonstrated by decreased Runx2 expression and ALP activity. Similar findings for the interaction between H19 and miR-140-5p were found in osteoarthritis and bone formation (Bi et al., 2020; Yang et al., 2020). Importantly, our work provides insight into the understanding of vascular calcification.

Our previous work and other reports have shown that miRNAs, such as miR-34b and miR-204, play a key role during the osteogenic differentiation of VSMCs (Lin et al., 2018; Lin et al., 2019). miR-140-5p has been reported to suppress osteoblast differentiation of mesenchymal stem cell by targeting osteogenesis marker gene BMP2 (Hwang et al., 2014). However, role of mir-140-5p in VSMC calcification remains unclear. In the present study, we found that inhibition of miR-140-5p increased while over-expression of miR-140-5p reduced the expression of Runx2 and ALP activity as well as Alizarin Red S staining. Notably, Satb2 was demonstrated to be the target of miR-140-5p in the regulation of VSMC calcification. Little has been clarified in the role of Satb2 in the regulation of vascular calcification. Recently, Hao et al. found that down-regulation of satb2 decreased VSMC calcification (Hao et al., 2016). But how Satb2 affect vascular calcification remains unclear. In the present work, we found that over-expression of Satb2 induced phosphorylation of both p38MAPK and ERK1/2, which indicates that Satb2 might regulate p38 and ERK1/2 signaling.

There exist some limitations for the present study. For example, all the study are performed *in vitro*, further study are needed to examine *in vivo* effects of H19 or miR-140-5p. Well-designed animal model might be helpful to demonstrate potential pro-calcification role of H19, which will provide evidence for potential drug targets to block arterial calcification.

In conclusion, our data indicate that H19 enhances osteoblastic differentiation of vascular smooth muscle cell by targeting miR-140-5p/Satb2 axis. Our study provides insight into understanding of vascular calcification and potential therapeutic targets for cardiovascular diseases.

## Data Availability

The original contributions presented in the study are included in the article/Supplementary Material, further inquiries can be directed to the corresponding author.
