# Supplementary material for: H19 Promotes Osteoblastic Transition by Acting as ceRNA of miR-140-5p in Vascular Smooth Muscle Cells
Source: Front Cell Dev Biol. 2022 Feb 7;10:774363. doi: 10.3389/fcell.2022.774363 (PMC8859097; doi:10.3389/fcell.2022.774363)
Supplement: Supplementary file 1 [file DataSheet1.pdf]

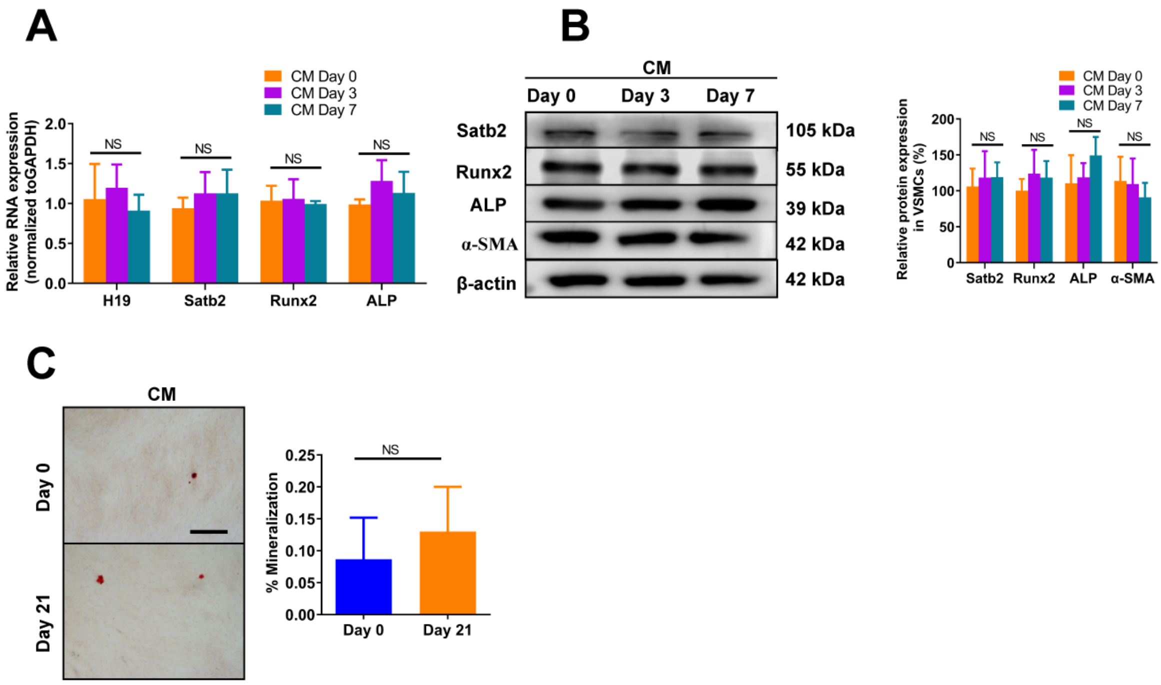

Supplementary Figure 1. No obvious change of calcification of VSMCs treated with conditional medium. VSMCs were treated with growth medium without β-glycerophosphate (β-GP) (CM medium) for 7days. (A) Expression of H19, calcification marker genes Runx2 and ALP, and Satb2 in VSMCs were determined by qRT-PCR. (B) Expression of Runx2, ALP, Satb2 and α-SMA were determined by Western blotting. The data were presented as densitometric ratios normalized to β-actin (B, right panel). (C) Alizarin Red S staining were measured in VSMCs incubated with CM for 21 days. Representative microscopic views are shown. Scale bar represents 200 μm. Data were presented as ratio of positive staining area (right panel). Each experiment was repeated for three times. The data represent the mean ± SD of triplicates. *p<0.05.

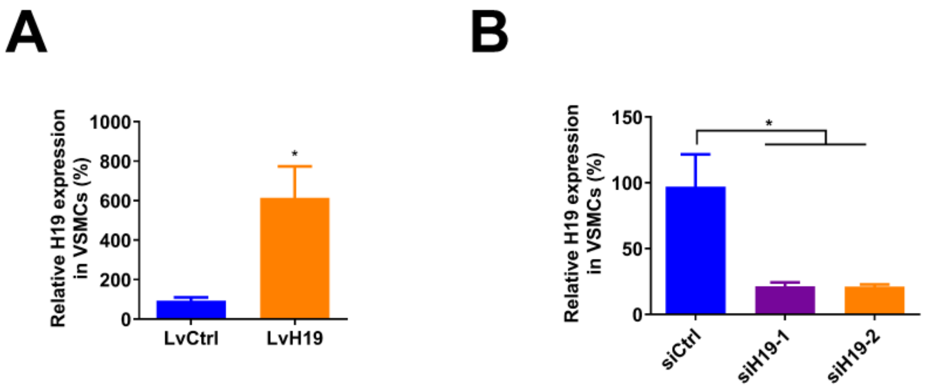

Supplementary Figure 2. Validation of H19 expression after overexpression or down-regulation. (A)VSMCs were treated with lentivirus containing full length of H19 cDNA (LvH19) or control cDNA (LvCtrl) and the efficacy was confirmed by qRT-PCR. (B) The expression of H19 in VSMCs after infected with H19 siRNA (siH19) or control siRNA (siCtrl) was evaluated by qRT-PCR. Each experiment was repeated for three times. The data represent the mean ± SD of triplicates. *p<0.05.

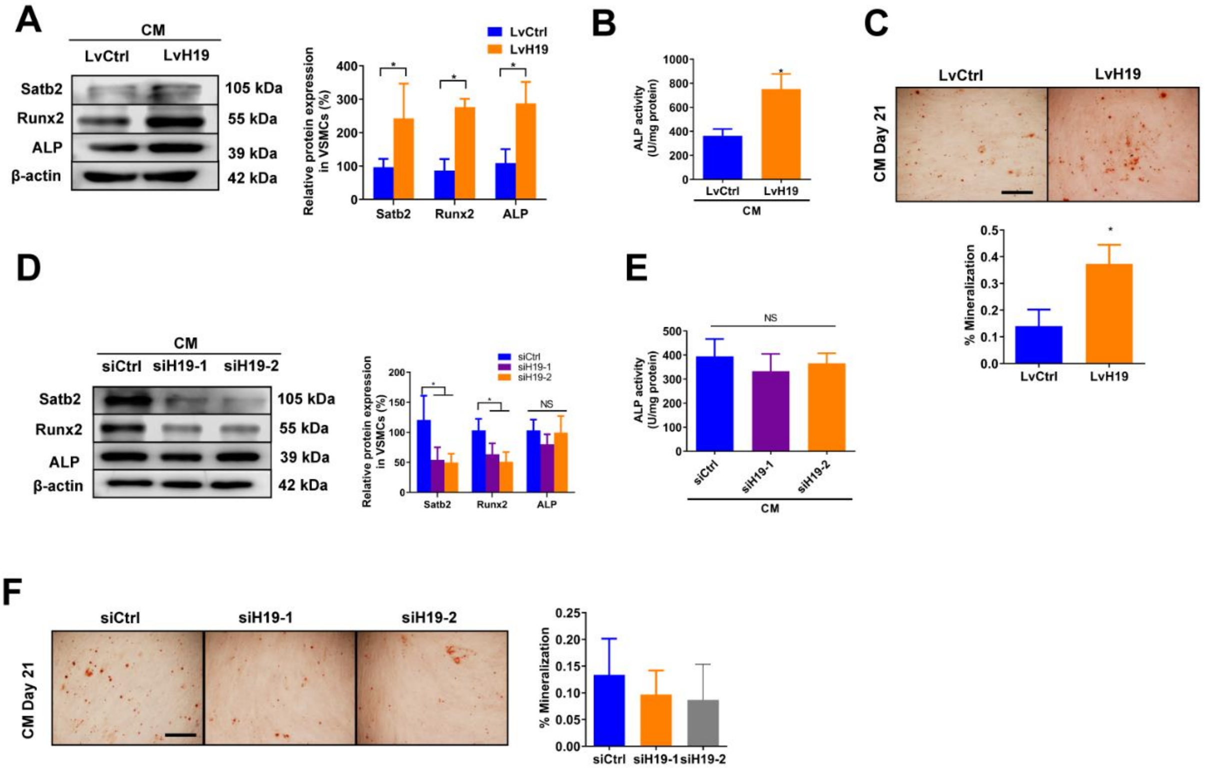

Supplementary Figure 3. H19 promote osteogenic differentiation and mineralization of VSMCs treated with CM medium. (A-C) VSMCs were treated with conditional medium (CM) along with either lentivirus containing full length of H19 cDNA (LvH19) or control cDNA (LvCtrl). The protein expression of Runx2, ALP, and Satb2 were determined by Western blotting in VSMCs after treatment for 2 days (A). Data were presented as densitometric ratios normalized to β-actin (A, right panel). The ALP activity was determined by an Elisa kit in VSMCs after treatment 2 days (B). Alizarin Red S staining were measured in VSMCs after treatment for 21 days. Representative microscopic views are shown (C). Scale bar represents 200 μm. Data were presented as ratio of positive staining area (C, lower panel). (D-F) VSMCs were incubated with OM medium along with either H19 siRNA (siH19) or control siRNA (siCtrl). The protein expression of Runx2, ALP, and Satb2 were determined by Western blotting after treatment for 2 days (D). Data were presented as densitometric ratios normalized to β-actin (D, right panel). The ALP activity was determined by an Elisa kit in VSMCs after treatment for 2 days (E). Alizarin Red S staining were measured in after

treatment for 21 days. Representative microscopic views are shown (F). Scale bar represents 200 μm. Data were presented as ratio of positive staining area (F, right panel). Each experiment was repeated for three times. The data represent the mean ± SD of triplicates. *$p<0.05$.
